# Predictive factors for entry to long-term residential care in octogenarian Māori and non-Māori in New Zealand, LiLACS NZ cohort

**DOI:** 10.1186/s12889-020-09786-z

**Published:** 2021-01-06

**Authors:** Marycarol Holdaway, Janine Wiles, Ngaire Kerse, Zhenqiang Wu, Simon Moyes, Martin J. Connolly, Oliver Menzies, Ruth Teh, Marama Muru-Lanning, Merryn Gott, Joanna B. Broad

**Affiliations:** 1 Department of Geriatric Medicine, University of Auckland, C/- Waitematā District Health Board, Takapuna, PO Box 93 503, Auckland, New Zealand; 2 School of Population Health, University of Auckland, Auckland, New Zealand; 3 Waitematā District Health Board, Auckland, New Zealand; 4 Auckland District Health Board, Auckland, New Zealand; 5 James Henare Māori Research Centre, University of Auckland, Auckland, New Zealand; 6 School of Nursing, University of Auckland, Auckland, New Zealand

**Keywords:** Long-term care, Advanced age, Risk factors, Care transition, Ethnic differences, Health services, Indigenous peoples

## Abstract

**Background:**

Long-term residential care (LTC) supports the most vulnerable and is increasingly relevant with demographic ageing. This study aims to describe entry to LTC and identify predictive factors for older Māori (indigenous people of New Zealand) and non-Māori.

**Methods:**

LiLACS-NZ cohort project recruited Māori and non-Māori octogenarians resident in a defined geographical area in 2010. This study used multivariable log-binomial regressions to assess factors associated with subsequent entry to LTC including: self-identified ethnicity, demographic characteristics, self-rated health, depressive symptoms and activities of daily living [ADL] as recorded at baseline. LTC entry was identified from: place of residence at LiLACS-NZ interviews, LTC subsidy, needs assessment conducted in LTC, hospital discharge to LTC, and place of death.

**Results:**

Of 937 surveyed at baseline (421 Māori, 516 non-Māori), 77 already in LTC were excluded, leaving 860 participants (mean age 82.6 +/− 2.71 years Māori, 84.6 +/− 0.52 years non-Māori). Over a mean follow-up of 4.9 years, 278 (41% of non-Māori, 22% of Māori) entered LTC; of the 582 who did not, 323 (55%) were still living and may yet enter LTC. In a model including both Māori and non-Māori, independent risks factors for LTC entry were: living alone (RR = 1.52, 95%CI:1.15–2.02), self-rated health poor/fair compared to very good/excellent (RR = 1.40, 95%CI:1.12–1.77), depressive symptoms (RR = 1.28, 95%CI:1.05–1.56) and more dependent ADLs (RR = 1.09, 95%CI:1.05–1.13). For non-Māori compared to Māori the RR was 1.77 (95%CI:1.39–2.23). In a Māori-only model, predictive factors were older age and living alone. For non-Māori, factors were dependence in more ADLs and poor/fair self-rated health.

**Conclusions:**

Non-Māori participants (predominantly European) entered LTC at almost twice the rate of Māori. Factors differed between Māori and non-Māori. Potentially, the needs, preferences, expectations and/or values may differ correspondingly. Research with different cultural/ethnic groups is required to determine how these differences should inform service development.

## Background

Despite attempts in many countries to reduce use of long-term residential care (LTC), utilisation is common with increasing age [1, 2]. LTC facilities provide accommodation and support for people whose needs for care surpass what is manageable at home with care/support from unpaid/paid carers. Terminology for residential aged care varies by country and level of care. Terms include nursing homes and geriatric hospital care, (for those with high dependency care needs); hostels, residential homes, rest homes, assisted living, (for those who need low dependency care needs,) and dementia care and psychogeriatric care. In this paper LTC refers to low, high, dementia and psychogeriatric care residential facilities, for both short and long-term care. Factors identified as predicting entry to LTC vary according to the availability of services and where and how studies are conducted [2].

Throughout the member countries of the OECD (Organisation for Economic Co-operation and Development), demand for LTC services is expected to increase because the number of people aged 85+ years is rising rapidly. For example, in Aotearoa New Zealand (NZ), official estimates expect numbers aged 85+ will triple, from 75,000 in 2013 to 230,040 by 2040 [3]. In NZ, over two-thirds of people reaching 85 years eventually move into a LTC facility [4]. Understanding service demand is important in countries that have a universal healthcare system such as NZ where all residents have access to healthcare without requiring private health insurance. International comparisons demonstrate more than ten-fold variation in use of LTC [5, 6] Utilisation is related to regional availability of facilities [7], specific disease states such as diabetes [8], differences by country/region and between cultural, religious, ethnic and/or racial groups [2]. In the USA, ethnic minorities and indigenous people enter LTC with poorer health than their European-majority counterparts [9]. Disparities in health outcomes of older indigenous people are often described [10–12] but seldom become a population-based research focus.

This study is drawn from *Life and Living in Advanced Age: A Cohort Study in New Zealand - Te Puawaitanga o Nga Tapuwae Kia Ora Tonu study* (LiLACS-NZ) which draws from a population-based sampling frame with indigenous (aged 80–90 years) and non-indigenous (aged 85 years) cohorts of similar size designed to provide similar statistical power. LiLACS-NZ aims to identify predictors of successful advanced ageing for Māori and non-Māori [13, 14] through acknowledging Māori methods and ideologies. Māori culture and Māori kaumātua (elders, both genders) are important components of the cultural fabric of NZ society. Kaumātua hold leadership roles in their whānau (extended family), hapū (sub-tribe), and iwi (tribe). They act as guardians of tikanga (Māori customs and practices) and are generally well respected in their communities [15]. In turn, whānau have cultural obligations to care for and support their kaumātua, sometimes at considerable personal cost [16].

Most commonly reported risk factors for LTC entry are those that constrain independent living: impaired mobility and inability to complete simple tasks such as washing, toileting, shopping, cooking and other ADLs are consistently identified [2]. Other relevant factors include living alone and recurrent falls, both associated with safety [17–19]. Older age is also a strong predictor of LTC [20], however few studies predicting LTC entry focus on the oldest old (80+) [21]. Importantly, risk factors for entry to LTC may differ for people aged 80+ compared to younger age groups. In a German study, cognitive and functional impairment predicted entry for younger old people (< 80 years) but not for those aged over 82 years [22]. In NZ, research on the topic was limited to small and/or focused studies, [23, 24] but recently a study by Jamieson et al. evaluating social factors influencing LTC entry in those receiving a support needs assessment, found that living alone, negative social interactions, perceived loneliness and carer stress were independently associated with higher likelihood of LTC admission [25]. Further, whether predictive factors for LTC entry apply similarly to all ethnic groups, or why some ethnic groups use LTC less often than others, is largely unaddressed.

Inequities in social determinants of health and healthcare access drive large health and mortality disparities between Māori and non-Māori [26, 27]. Currently, the majority of LTC residents in NZ are European, with few older Māori residents. In 2013, 5.6% of the population aged 65+ were Māori, and 87.8% were European [28]. Of those in LTC, however, just 3.3% were Māori, with 93.4% European [29]. In the LiLACS-NZ study, baseline data showed that, of those with frequent and high levels of need for care, Māori were less likely to be living in LTC [30, 31]. Further, in the study of carers of LiLACS-NZ, Māori received significantly more hours of informal (unpaid) care e.g. from family, than non-Māori, and Māori carers were more likely to live in the same house or on the same property [30]. Living in multi-generational family settings means they are less likely to qualify for formal home support (e.g. help with shopping, travel for medical attention, or household cleaning), thus perversely leading to further disparities in access to formal supports for Māori. Indeed, access to formal home support at baseline in LiLACS-NZ also differed, with Māori receiving formal care being discernably more disabled than those not receiving formal care, a distinction not holding for non-Māori [31]. Whānau has traditionally been an integral part of health for Māori, and indeed the popular Te Whare Tapa Whā [32] model of Māori health notes whānau as one of the four mainstays of health for Māori. One of the recognised roles of whānau is that it “looked after its own aged or disabled members” [33]. How these differences in care patterns and cultural priorities may play out when accessing LTC is the subject of this report.

In line with other countries, NZ urgently needs better understanding of health, demographic and socio-cultural factors that explain ethnic differences in healthcare use, including entry to LTC. The main aims of this prospective study using the LiLACS-NZ cohorts were to assess the proportion who entered LTC over the study period and to identify independent risk factors for LTC entry among NZ Māori and non-Māori octogenarians.

## Methods

### Study design

LiLACS-NZ is a bicultural cohort study of people in advanced age, by design enrolling non-Māori in their 85th year, and Māori aged 80–90 years [13, 34]. Geographical boundaries of the District Health Boards (DHB) of the Bay of Plenty and northern part of the Lakes areas were chosen as having a stable population, with mixed urban and rural populations including Māori communities. General practitioners and Hauora Māori (Māori health services) provide primary healthcare, while secondary care services are provided by public hospitals in two cities and one town. Local municipal councils have no direct role in health service or LTC provision.

At baseline, there were 421 (45%) Māori and 516 (55%) non-Māori [13, 34]. Study participants completed full (long) or core (short) interviews annually from 2010 until 2016, death, or dropout. Participants who were unable to complete a full interview due to illness or other restrictions completed the core interview. Most participants also completed medical reviews, blood tests and physical assessments yearly. Participants who chose the core interview did not have a physical assessment or blood tests.

LiLACS-NZ was guided by the Te Rōpū Kaitiaki o Ngā Tikanga Māori (Protectors of Principles of Conduct in Māori Research, Māori guidance group) who advised on interview content, conduct, etiquette and translation [13]. Interviews and physical assessments were undertaken by trained lay interviewers and nurses respectively, using standardised procedures. Primary care medical records and secondary care hospital records were accessed with consent [13, 35].

### Entry to LTC

The endpoint of LTC entry was defined as any reported transition into lower-level (rest home or specialist dementia care) or higher-level care (private hospital or psychogeriatric care), at any time after baseline interview. With no single authoritative information source available, to maximise completeness, LTC entry was established from any one or more of six sources: 1) place of residence from six waves of LiLACS-NZ interviews; 2) late life residence or place of death in LILACS-NZ end-of-life interviews; 3) discharge destination in Ministry of Health (MoH) hospitalisation discharge data; 4) receipt of long-term care subsidy from Contracted Care Payment System (CCPS) subsidy data; 5) healthcare needs assessment conducted while resident in LTC from interRAI (international Resident Assessment Instrument) Long Term Care Facility (LTCF) assessments [36]; and 6) place of death from the MoH National Mortality Collection Registry data. Follow up was from study baseline in 2010 to December 2016. Because of data limitations, both short- and long-term stays were included as LTC, though short-term residential care is rarely used in NZ. For a comprehensive explanation of the primary data sources (see Additional file 1).

### Risk factors for LTC entry

Potential predictive factors investigated were identified from the baseline questionnaires, primary care medical record review, blood test results and physical assessment [13]. Following discussion among authors and literature review, important factors for investigation were identified and where possible included in the investigation. Due to data limitations, factors reported from both core and full questionnaires only were included. Gender, marital status, and living arrangement were established at cohort inception by self-report. Ethnicity was established by self-identification using the NZ census question 2006 [37]. Self-rated health was from a single question in the SF-12 [38], falls were by self-report, and ADL function was from 11 questions from the NEADL scale [39] relating to the following areas: mobility, activities in the kitchen, domestic tasks, leisure activities and basic tasks (personal care, toileting, moving in and out of bed). Depressive symptoms were derived from the GDS [40] with a threshold ≥5 and/or from depressive symptoms (see Additional file 1). Those factors significantly associated with the endpoint in unadjusted exploratory logistic regressions were investigated in adjusted models.

### Statistical analysis

Scaled rectangle diagrams were used to visualise primary data sources of LTC transition events captured, and the overlap between them [41]. T-tests and chi-square tests were conducted to assess the association of LTC entry and individual predictive factors. Variables that were significant (*p* value ≤0.05) in univariate analyses and/or important in international studies were modelled for the whole cohort, and in separate models for Māori and non-Māori. Multivariable log-binomial regressions were used to identify significant independent predictive factors for entry to LTC and estimate the associated relative risk (RR) [42]. The modelled R^2^ assessed goodness-of-fit [43].

Analyses were performed using SAS version 9.4 (SAS Institute Inc., Cary, NC, USA) or R (R package version 3.3.1.). Significance testing used two-sided tests, with *p* ≤ 0.05 considered significant.

## Results

### Baseline characteristics

**Fig. 1:**
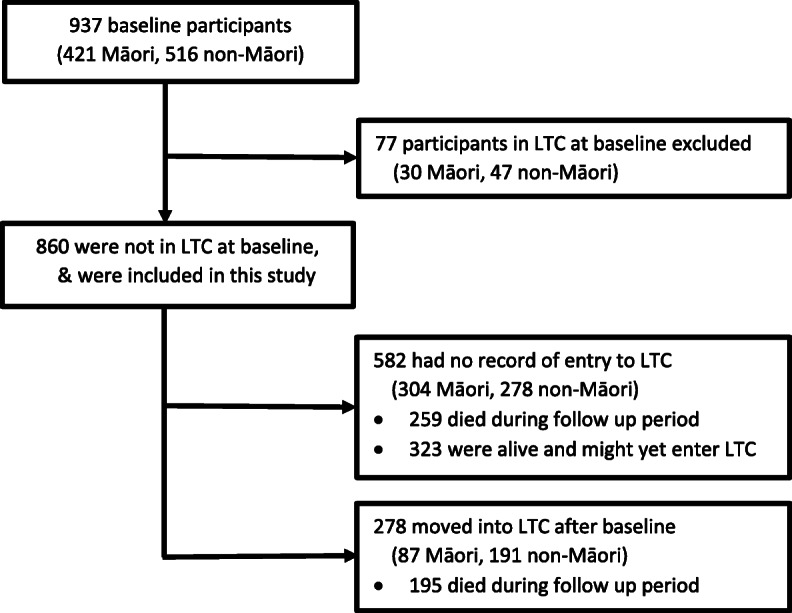
Entry to LTC study flowchart

Of 937 people recruited and interviewed, 77 were already in LTC at baseline (30 Māori, 47 non-Māori) and were excluded from analyses (Fig. 1). Of the 860 Māori and non-Māori remaining, 55% were female, with 62% not married (i.e. single/widowed) (Table 1). Mean age was 82.6 (Standard Deviation (SD) 2.71) years for Māori and 84.6 (SD 0.52) years for non-Māori. At baseline, at least 40% were living with others, 43% reported very good/excellent health, 18% reported falling ≥2 times per year; with help needed to perform 1.4 ADLs on average (Table 1).

**Table 1:** Baseline participant characteristics for All, Māori and non-Māori participants living at home

	All participants ***N*** = 860	Māori ***N*** = 391	Non-Māori ***N*** = 469
Age, mean (SD)	83.7 (2.12)	82.6 (2.71)	84.6 (0.52)
Women, n (%)	472 (55)	223 (57)	249 (53)
Not-married, n (%)	534 (62)	265 (68)	269 (57)
Living situation, n (%)			
Alone	296 (34)	109 (28)	189 (40)
With others	348 (40)	156 (40)	192 (41)
Unknown (Core questionnaire)	214 (25)	126 (32)	88 (19)
Self-rated health, n (%)			
Poor/fair	178 (21)	83 (21)	95 (20)
Good	313 (36)	141 (36)	172 (37)
Very good/excellent	369 (43)	167 (43)	202 (43)
Depressive symptoms, n (%)	246 (29)	139 (36)	110 (23)
Two or more falls in last 12 months, n (%)	158 (18)	68 (17)	90 (19)
ADLs, mean (SD)	1.41 (1.95)	1.58 (2.35)	1.27 (1.52)

Note: Participants in LTC at baseline interview (*n* = 77) are excluded. Depressive symptoms is indicated if participant’s Geriatric Depression Scale score ≥ 5 or where participant reports feeling depressed recently. ADLs is a count of 11 tasks participants did not perform independently from the Nottingham Extended Activities of Daily Living scale. SD = standard deviation

### Deaths and entry to long-term care

**Fig. 2:**
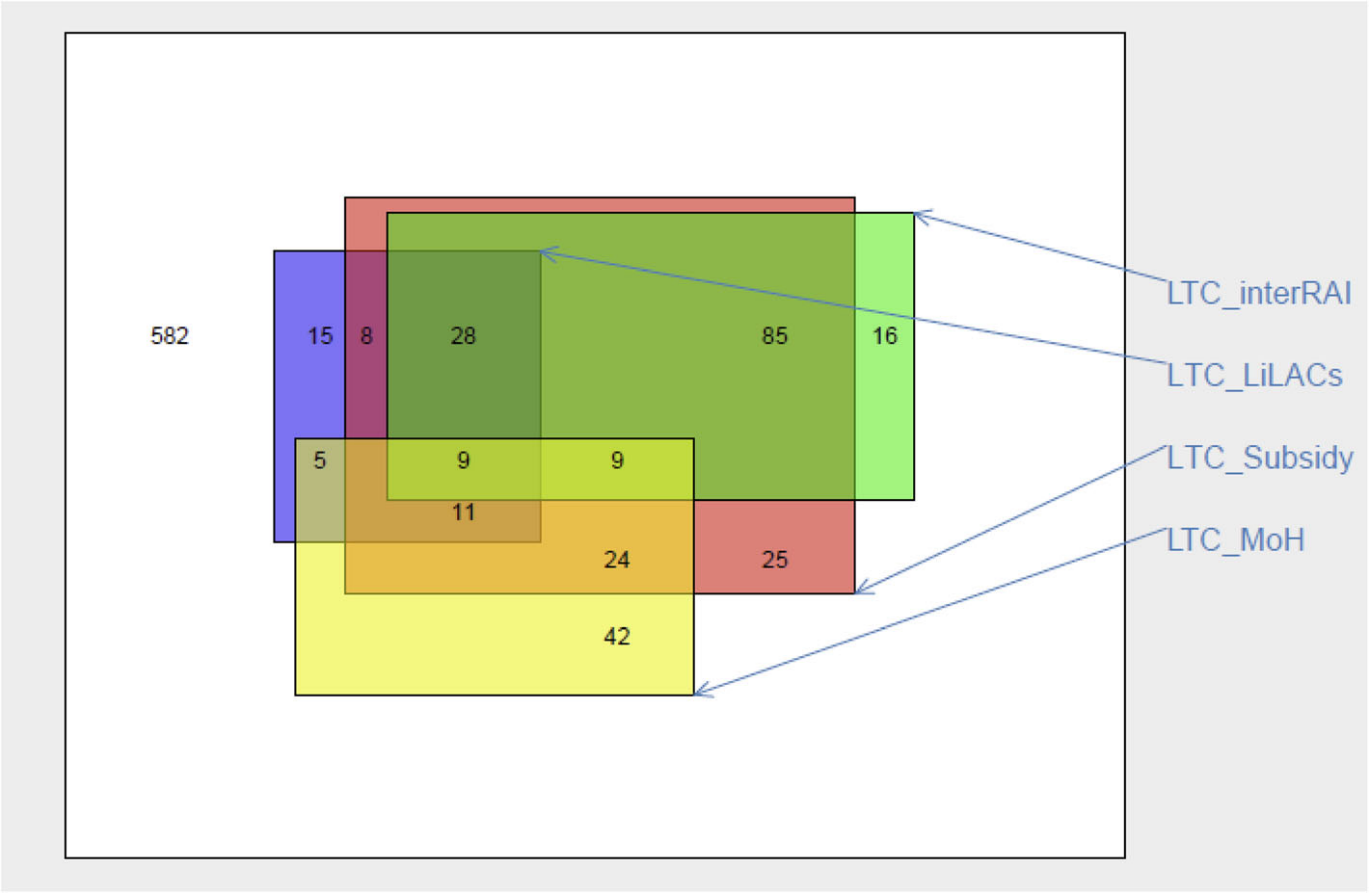
Entry to long-term care by source of information. Scaled rectangle diagram demonstrating LTC entry during follow-up, as captured by each of four data sources, showing their intersections. LTC_interRAI indicates an interRAI assessment conducted in/for a LTC facility. LTC_LiLACS represents LTC entry recorded in any LiLACs_NZ interview. LTC_Subsidy indicates government subsidy payments made for this person. LTC_MoH represents a LTC entry recorded in Ministry of Health hospitalizations data and/or National Mortality Collection Registry data

When all information sources were combined over a mean follow-up time of 4.9 years, 454 (53%) died (222 Māori, 232 non-Māori). One or more records of a transition to LTC was found for 278 (32%): 87 (22%) Māori and 191 (41%) non-Māori (Table 2, *p* < 0.0001). LiLACS-NZ interviews in waves 2–6 captured 76 (27%) of the 278 known transitions to LTC after baseline (Table 2). One or more MoH sources yielded almost all (*n* = 275) transitions, though no single source found more than 7 in 10 transitions; few were sourced from hospital discharge destination data (Fig. 2). No LTC indication was found for 582 participants and of these, 45% died during the follow-up period (154 of 304 Māori, 51%, and 105 of 278 non-Māori, 38%, *p* = 0.002).

**Table 2:** Death and Entry to long-term care in LiLACS NZ cohort, by source of information

	All Participants ***N*** = 860	Māori ***N*** = 391	Non-Māori ***N*** = 469
**Follow-up time, mean years (SD)**	**4.86 (1.94)**	**4.65 (1.87)**	**5.03 (2.00)**
**Died during follow-up, n (%)**	**454 (53)**	**222 (57)**	**232 (49)**
**Entry to LTC, n (%)**	**278 (32)**	**87 (22)**	**191 (41)**
No entry to LTC, n (%)	582 (68)	304 (78)	278 (59)
LiLACS NZ interviews, n (%)	76 (9)	23 (6)	53 (11)
Waves 2–6 participant interviews	63 (7)	18 (5)	45 (10)
End of Life Interviews	23 (3)	9 (2)	14 (3)
Ministry of Health sources, n (%)	275 (32)	84 (21)	191 (41)
CCPS subsidy	199 (23)	60 (15)	139 (30)
interRAI assessments	148 (17)	44 (11)	104 (22)
Mortality registry	90 (10)	31 (8)	59 (13)
Hospital discharge	26 (3)	7 (2)	19 (4)

Note: 77 participants (30 Māori, 47 non-Māori) who were resident in LTC at baseline were excluded

More than one source may report LTC entry for any participant. SD = standard deviation

Unadjusted associations of potential predictive factors for entry to LTC showed all variables in Table 1, except gender, were significantly associated with LTC entry either for Māori, non-Māori, or when combined (Table 3). For Māori, statistically significant variables were age, self-rated health, depressive symptoms and dependency in ADLs, and for non-Māori, marital status, living situation, self-rated health, depressive symptoms, falls and dependency in ADLs (Table 3).

**Table 3:** Entry to long-term care by baseline characteristics, unadjusted comparisons

	All Participants			Māori			non-Māori		
	no LTC ***n = 582***	LTC ***n = 278***	***P*** value	no LTC ***n = 304***	LTC ***n = 87***	***P*** value	no LTC ***n = 278***	LTC ***n*** = ***191***	***P*** value
**Ethnicity, n (%)**									
Māori	304 (52)	87 (31)	**< 0.001**	304 (100)	87 (100)		–	–	–
Non-Māori	278 (48)	191 (69)		–	–		278 (100)	191 (100)	
**Age, mean (SD)**	83.4 (2.2)	84.2 (1.8)	**< 0.001**	82.4 (2.6)	83.2 (2.9)	**0.01**	84.6 (0.5)	84.6 (0.5)	0.44
**Gender, n (%)**									
Women	318 (55)	154 (55)	0.83	175 (58)	48 (55)	0.69	143 (51)	106 (55)	0.39
Men	264 (45)	124 (45)		129 (42)	39 (45)		135 (49)	85 (45)	
**Marital status, n (%)**									
Not-married	350 (60)	184 (66)	0.09	204 (67)	61 (70)	0.60	146 (53)	123 (64)	**0.01**
Married	232 (40)	94 (34)		100 (33)	26 (30)		132 (47)	68 (36)	
**Living situation, n (%)**									
Alone	183 (31)	115 (41)	**0.001**	78 (26)	31 (36)	0.16	105 (38)	84 (44)	**0.001**
With others	259 (45)	89 (32)		127 (42)	29 (33)		132 (47)	60 (31)	
Unknown **(Core)**^**a**^	140 (24)	74 (27)		99 (33)	27 (31)		41 (15)	47 (25)	
**Self-rated health, n (%)**									
Poor/fair	96 (16)	82 (29)	**< 0.001**	56 (18)	27 (31)	**0.04**	40 (144)	55 (29)	**< 0.001**
Good	218 (37)	95 (34)		113 (37)	28 (32)		105 (38)	67 (35)	
Very good/excellent	268 (46)	101 (36)		135 (44)	32 (37)		133 (48)	69 (36)	
**Depressive symptoms, n (%)**									
Yes	147 (25)	99 (36)	**0.002**	97 (32)	39 (45)	**0.03**	50 (18)	60 (31)	**< 0.001**
No	435 (75)	179 (64)		207 (68)	48 (55)		228 (82)	131 (69)	
**No. of falls in last 12 months, n (%)**									
2+ falls	91 (16)	67 (24)	**0.003**	49 (16)	19 (22)	0.21	42 (15)	48 (25)	**0.007**
0,1 fall	491 (84)	211 (76)		255 (84)	68 (78)		236 (85)	143 (75)	
**ADLs, mean (SD)**	1.21 (1.85)	1.82 (2.08)	**< 0.001**	1.45 (2.33)	2.03 (2.38)	**0.04**	0.96 (1.04)	1.72 (1.93)	**< 0.001**

Note: Participants in LTC at baseline interview (n = 77) are excluded. Continuous variables are reported as mean (SD); categorical variables are reported as n (%). Depressive symptoms is indicated if Geriatric Depression Scale score ≥ 5 or where participant reports feeling depressed recently. ADLs is a count of 11 tasks participants did not perform independently from the Nottingham Extended Activities of Daily Living scale. *P* < 0.05 is indicated in bold. SD = standard deviation

^**a**^ The relationship between completing the LiLACS NZ core questionnaire and LTC entry is examined as a variable in living situation because participants who completed a core questionnaire had missing data about living situation

Multivariable models included all significant variables identified from the univariate analyses, then falls was removed as non-contributing to the model fit. In the multivariable model of all 860 participants living in the community at baseline, non-Māori had almost twice the likelihood of LTC entry than Māori (RR = 1.77, 95%CI:1.39–2.23, *p*-value < 0.001, Table 4). Reporting *poor*/*fair* health compared to *very good/excellent* health, and depressive symptoms compared to not, were also associated with greater likelihood of LTC entry (RR = 1.40 and 1.28, respectively).

**Table 4:** Multivariable models of baseline characteristics to predict LTC entry

	All participants ***N*** = 860	Māori ***N*** = 391	Non-Māori ***N*** = 469
	RR (95% CI) ***p***-value	RR (95% CI) ***p***-value	RR (95% CI) ***p***-value
Non-Māori (vs Māori)	1.77 (1.39, 2.23) **< 0.001**	–	–
Age (each year of age)	1.06 (1.00, 1.12) 0.07	1.08 (1.01, 1.15) **0.02**	0.99 (0.80, 1.21) 0.90
Men (vs women)	1.01 (0.83, 1.23) 0**.**89	1.10 (0.75, 1.63) 0.62	1.01 (0.81, 1.26) 0.94
Not-married (vs married)	1.00 (0.77, 1.29) 0.97	0.86 (0.55, 1.34) 0.50	1.10 (0.79, 1.54) 0.56
Living situation			
Alone (vs with others)	1.52 (1.15, 2.02) **0.004**	1.72 (1.06, 2.80) **0.03**	1.41 (0.99, 2.03) 0.06
Unknown^a^ (vs with others)	1.31 (1.00, 1.71) 0**.**05	1.07 (0.67, 1.71) 0.76	1.41 (0.98, 2.04) 0.07
Self-rated health			
Poor/fair (vs v.good/excellent)	1.40 (1.12, 1.77) **0.004**	1.53 (0.98, 2.41) 0.06	1.38 (1.06, 1.80) **0.02**
Good (vs v.good/excellent)	1.04 (0.82, 1.30) 0.77	1.05 (0.66, 1.68) 0.84	1.02 (0.78, 1.33) 0.87
Depressive symptoms (vs not)	1.28 (1.05, 1.56) **0.01**	1.38 (0.95, 2.02) 0.09	1.18 (0.93, 1.49) 0.17
ADLs (each additional ADL)	1.09 (1.05, 1.13) **< 0.001**	1.04 (0.97, 1.12) 0.26	1.14 (1.09, 1.19) **< 0.001**
R^2^	0.11	0.06	0.10

Note: Participants in LTC at baseline interview (n = 77) are excluded. Depressive symptoms indicates a Geriatric Depression Scale score ≥ 5 or where participant reports feeling depressed recently. ADLs is a count of 11 tasks participants did not perform independently from the Nottingham Extended Activities of Daily Living scale. *P*-values < 0.05 are indicated in bold

^a^People with missing data about living situation were those who completed a Core questionnaire

### Predictive factors for entry to LTC for Māori & non-Māori

In the multivariable model of Māori participants only, older age and living alone were significant independent predictors of admission to LTC (Table 4). In the multivariable model for non-Māori only, *poor/fair self-rated health* and *greater dependency in ADLs* were significant independent predictors of admission to LTC.

Differences in significant predictive factors were noted: living alone status was associated with greater likelihood of entry to LTC among Māori (RR = 1.72, 95%CI:1.06–2.80) but did not reach significance for non-Māori (RR = 1.41, 95%CI:0.99–2.03); greater functional dependence at baseline was important for non-Māori (RR = 1.14, 95%CI:1.09–1.19) but not Māori. Measures of model fit showed that the three models had only modest fit (R^2^ = 0.06 to 0.11, Table 4).

## Discussion

This is the first study to examine prospectively the likelihood of LTC placement for a large group of very old indigenous people. Within the context of advanced ageing in Aotearoa NZ, we show that LTC is used less frequently by indigenous peoples (Māori) and also that the risk factors differ between the indigenous and non-indigenous peoples. These findings add to both the international evidence regarding factors leading to LTC placement [2] and to the dialogue on health services for indigenous peoples [11]. It is evident that non-Māori and Māori in NZ use LTC in different ways, possibly partly because family and whānau support for older family members differs between indigenous and non-indigenous New Zealanders.

Likelihood of entry to LTC for non-Māori is approaching double that for Māori when adjusted for other factors: age, gender, marital status, living situation, self-rated health, depressive symptoms and functional status. Furthermore, in ethnic specific models, Māori and non-Māori had different profiles of factors associated with subsequent LTC entry. For example, living alone at baseline predicted LTC entry for Māori but not for non-Māori, and dependency in ADLs was associated with subsequent LTC entry among non-Māori but not among Māori. This is contrary to most other studies where functional limitation is found to be the primary driver of transition [2]. Other factors, measured and/or unmeasured, may also play a role.

Cultural perspectives are relevant to the search for reasons for the observed differences. That fewer Māori entered LTC over almost 5 years of follow-up may illustrate the impact of Māori cultural practices of whanaungatanga (family connection), manaakitanga (caring for and respecting others) and āwhina (supporting others) whereby whānau have obligations and responsibilities to care for their kaumātua. Those cultural expectations to provide informal support may contribute to fewer Māori receiving formal home-based services (despite worse health), more common use of informal care and less use of LTC, by Māori [31]. Living alone was a stronger factor in LTC placement for Māori than non-Māori, suggesting that the impact of whānau and family care meant for some Māori entry to LTC was delayed or avoided. Anecdotal reports of Māori whānau members moving house (and country) specifically to provide informal care to support kaumātua could not be examined in this analysis, however, these cultural obligations and observed differences in patterns of care, as in baseline data [30, 31, 44], are likely to be relevant to the observed difference in rate of admission to LTC. However, the extent to which Māori whānau will continue to find capacity to provide care into the future is unclear given changing family structures and increased care demands due to rising numbers of kaumātua and their increasing longevity [28]. Older Māori are also less able to access primary care services due to the cost of primary care in NZ [45], thus community support for Māori may need to be strengthened.

We are also unable, in this analysis, to establish whether differential access to home-based supports or culturally driven preferences are the main drivers of LTC entry. While culture-related care patterns may contribute to the lack of association between ADL level and entry to LTC among Māori, other questions are also likely to be relevant. Do Māori who enter LTC find the environment (staff, other residents, systems, food, buildings and gardens) inclusive and accepting of non-European traditions, values and practices? Are LTC institutions able to support and embrace Māori tikanga appropriately? For people in advanced age of any ethnicity, how can family/whānau who provide home-based informal care, often at the loss of careers and income [30, 46] be better supported with health service navigation, skill development (for formal and informal carers) and/or financial assistance [47]. Public discussion is urgently needed regarding access and appropriateness of support services for people of advanced age whether in LTC settings or at home.

There are three additional learnings from this study. Firstly, and consistent with international data, depressive symptoms (as measured by western tools), predicts LTC entry for all participants [48, 49]. Depression has been recognised as sufficient to overwhelm an older person and their support network [50], resulting in escalation in need and entry to LTC [48]. Jamieson et al. confirmed this for NZ in a large study of a higher risk group than the current study, and in which Māori were only half as likely to enter LTC [25]. Risk of entry was 13% higher when the person was depressed, and 28% higher when the carer scored high on a measure of carer stress [25]. Primary care practitioners, practice nurses and community members could be encouraged to recognise and address depressive symptoms in order to avert escalation to LTC, but research is needed to better understand depressive symptoms and coping mechanisms in different cultural settings.

Secondly, supporting a recent systematic review [2] and other NZ research [25], and despite the preponderance of women in care facilities [29], neither gender was at greater likelihood of LTC entry once adjusted for other factors. Greater numbers of women in LTC may be explained instead by women often being married/partnered to older men who die before them, then because they live longer, they stay longer in LTC [29], and also by other factors correlated to gender but where numbers were inadequate to reach significance in the models. Gender was not a significant factor associated with receipt of services in the LiLACS-NZ study after adjustment for living arrangement and marital status [31]. Nevertheless, international studies suggest that some factors are more important for men and women, respectively. For example, being unmarried, living alone and incontinence are associated with greater likelihood of entry to LTC among men, while functional impairment carries a higher likelihood among women [2]. Elsewhere, the association between LTC entry and widowhood is linked to gender and is time-dependent, particularly within the first month of widowhood when older men are at greater likelihood of LTC entry [51, 52].

Thirdly, this study demonstrates that in NZ, no single information source could identify all participants who enter LTC. This is previously reported [53], and continues to have repercussions for monitoring public health and service utilisation. In the current study the most complete source was the subsidy data, finding 199/278 (69%) of those who entered LTC during the study period. However Government subsidies are available only for residents who are deemed eligible for financial assistance after a care needs assessment and an assessment of their assets. Those in lower-level care paying entirely privately are thus not captured from subsidy data and are often omitted from utilisation reports [53]. Some under-reporting is thus possible even using six different data sources. InterRAI data, although introduced after the study start, were next most complete, however the source contributing the most additional information was the death registry data. In all, the three-way combination of subsidy, place of death and hospital discharge data found 247/278 (89%) of those recognised as entering LTC.

### Limitations

There are several limitations of this study. The numbers who entered LTC may be underestimated because interRAI assessments were introduced after study inception, and place of death and residence in the death registry data were accessed only until December 2016. The study was conducted in only one region of NZ. The variables examined were restricted to characteristics collected in the core questions in the LiLACS-NZ baseline questionnaire with one exception, living situation, which was in the full questionnaire only. In consequence, receipt of supports (informal or formal) at baseline, blood results and physical assessment were too incomplete for modelling. Changes occurring after baseline and gathered in waves 2–6, such as having a stroke or losing a spouse, were not included in the models. Nor was time to entry to LTC considered in competing risks models [54] because date of entry to LTC was not available. The study does not distinguish between type of LTC stay, for example long- or short-term, high- or low-level, palliative or dementia care, and nor were individual preferences, family expectations or values considered. Future research is needed to examine predictive factors for separate levels of care and related changes over time, as well as to examine participant characteristics which trigger immediate entry to LTC. Finally, fewer Māori, together with the smaller proportion of Māori living alone [55, 56] and the greater proportion of data missing for Māori (because of higher use of the core questionnaire) may have reduced the power of the study and made type II errors more likely.

## Conclusions

Non-Māori people age over 80 years entered LTC at almost twice the rate of Māori during the 6 years of follow-up in this study, after adjustment for important correlates. Factors associated with LTC entry differed between Māori and non-Māori participants, with factors among non-Māori being similar to those identified in overseas studies. For our indigenous peoples, factors other than disability were important and this finding may be related to cultural choices and values. Informed discussion about access to and appropriateness of support services for older people and their carers – in LTC settings and at home – is needed.

## Supplementary Information

**Additional file 1.** Primary data sources. Describes the data sources used for the risk factors and the endpoint of interest, i.e. entry to LTC.

## Data Availability

The datasets generated and/or analysed during the current study are not publicly available due to the personal and sensitive nature of some of the information, and because no consent was obtained from participants at the time the study commenced. Bona fide researchers who have a research question and wish to make use of the data should contact the corresponding author in the first instance. Publicly available data, including hospitalisations, subsidy payments, interRAI assessments and death registration records may be obtained through the appropriate government sources following ethics and approval processes.

## Abbreviations

95%CI: 95% confidence interval
ADL: Activity of Daily Living
CCPS: Contracted Care Payment System
GDS: Geriatric Depression Scale
HC: interRAI Home Care Form
interRAI: international Resident Assessment Instrument
LiLACS-NZ: Life and Living in Advanced Age, a Cohort Study in New Zealand
LTC: Long-term care
LTCF: interRAI Long Term Care Facility Form
MoH: Ministry of Health
NEADL: Nottingham Extended Activities of Daily Living Scale
NZ: New Zealand
OECD: Organisation for Economic Co-operation and Development
RR: Relative risk
SD: standard deviation
