# Supplementary material for: Predictive factors for entry to long-term residential care in octogenarian Māori and non-Māori in New Zealand, LiLACS NZ cohort
Source: BMC Public Health. 2021 Jan 6;21:34. doi: 10.1186/s12889-020-09786-z (PMC7788817; doi:10.1186/s12889-020-09786-z)
Supplement: Supplementary file 1 — Additional file 1. Primary data sources. Describes the data sources used for the risk factors and the endpoint of interest, i.e. entry to LTC. [file 12889_2020_9786_MOESM1_ESM.docx]

## Supplementary LiLACS Material: Data sources

### Primary data sources of transition to LTC

1. Full and Core LiLACS NZ interviews (all waves): Long-term care (LTC) entry was counted when type of residence was recorded as *rest home/private hospital.*
2. LiLACS NZ End of Life (EOL) interviews (all waves): LTC entry was counted when the home or place of death was reported as *rest home* or *private hospital,* or they (deceased) spent time in their last 3 months of life in a *rest home/private hospital,* as reported by a family/whānau member.
3. Ministry of Health (MoH) hospitalisations data: LTC entry was counted when the treatment facility code following discharge indicates treatment in LTC, and date of discharge is after the baseline interview.
4. Contracted Care Payment System (CPSS) subsidy data: LTC entry was counted when there is record subsidy payment from the respective DHB, after the date of the baseline interview. The CPSS database contains all people who receive publicly funded residential care and approximately half of privately funded LTC residents.
5. interRAI assessment data: LTC entry was counted when participants received a LTC facility (LTCF) assessment while resident in a LTC facility, and the admission date occurs after the baseline interview or when any date of entry to LTC is recorded in interRAI after baseline interview. LTCF assessments were completed in each quarter year from 2012 onward, but not prior.
6. MoH National Mortality Collection Register: LTC entry was counted when place of death indicates LTC entry*.* Participants who died in a retirement village with an LTC facility annexed were not counted. Mortality data from the period up to December 2016 was collected.

### Long-term care risk factors

#### Ethnicity, age, gender

Ethnicity was established by self-identification using the NZ census question 2006.^[[1]](#endnote-1)^ Participants reported which *ethnic group(s)* [they] *belong to,* by responding to a list of options including NZ European, Māori, Samoan, Cook Island Māori, Tongan, Niuean, Chinese, Indian, other European or other. Participants who responded *Māori* were assigned the Māori group irrespective of other ethnic groups also reported. All other participants were assigned to *non-Māori*. Age was derived from the date of birth provided at commencement of the baseline interview. Participants reported their gender and two options were offered, male or female.

#### Marital status

Responses concerning current marital status were dichotomized into class levels: *married (including partnered)* or *not married* (including *never married/partnered, widow/widower, separated, divorced).*

#### Living Situation, core questionnaire and missing data

Participants who completed a full LiLACS NZ baseline interview reported *who* [they] *live with most of the time* from a selection panel including *alone, with spouse/partner only, with spouse and child/other relative, with spouse and non-relatives, with child (not spouse), with other(s) not spouse or children* – the last of which they were asked for specification. Dichotomized class levels representing those *living alone* and those *living with others* were derived.

Participants who completed a core questionnaire were not asked the question about living situation thus there is missing data for this group. This group of participants is represented by variable level *Core Questionnaire.* In order to avoid interactions in the model, living situation was the only risk factor asked exclusively of participants taking the full interview included in this analysis.

#### Self-rated health

Self-rated health was from a single question in the SF-12.^[[2]](#endnote-2)^ Three class levels for self-rated health: *excellent/very good, good, fair/poor,* were derived from five possible responses with similar headings: *one (poor), two (fair), three (good), four (very good)* and *five (excellent).*

#### Depressive symptoms

Participants who took the full questionnaire and scored ≥5 on the Geriatric Depression Scale (GDS)^[[3]](#endnote-3)^ were classified as having *depressive symptoms*. Participants who took the core Questionnaire and reported they were *bothered by feeling down, depressed or hopeless in the past month* were also assigned this level*.* Combining these into one response is justified because the questions provide similar information about participant outcomes (correlation not reported).

#### Blood tests

#### Anaemia and diabetes detected in blood tests at baseline were considered for analyses, but incomplete data meant they were not made available for multivariate modelling.

#### Falls

Falls were self-reported by participants responded to a question concerning *how many times* [they] *have fallen in the past 12 months* and responded accordingly with one of the following: *none, one, two or three, four or more.* Variable levels grouped participants who reported fewer than 2 falls, or 2 or more falls.

#### Functional status & Activities of Daily Living

ADL function was from the Nottingham Extended Activities of Daily Living (NEADL) scale.^[[4]](#endnote-4)^ Eleven selected NEADL questions were asked on the core and 22 on the full interviews. The 11 included in the core were selected according to LiLACS NZ protocols and address the following areas: a) Mobility – *do you walk around outside?* b) Kitchen – *do you manage to feed yourself? Do you manage to make yourself a hot drink? Do you take hot drinks from one room to another? c)* Domestic tasks *– do you do your own housework? Do you do your own shopping? Do you do a full clothes wash?* d) Leisure activities – *do you use the telephone?* e) Other – *do you manage your personal care (brushing teeth and hair, washing your hands and face)? do you manage to go to the toilet? do you get in/out of bed?* Participants responded they could perform tasks *with help*, or *on my own/on my own with difficulty*. ADLs not completed independently were counted from 0-11.

1. Statistics New Zealand. National Ethnic Population Projections: 2013(base)-2038. Wellington Statistics New Zealand 2015. [↑](#endnote-ref-1)
2. Brazier JE, Roberts J. The estimation of a preference-based measure of health from the SF-12. Med Care 2004:851-59. [↑](#endnote-ref-2)
3. Sheikh JI, Yesavage JA. Geriatric Depression Scale (GDS): recent evidence and development of a shorter version. Clinical Gerontologist: The Journal of Aging and Mental Health 1986 [↑](#endnote-ref-3)
4. Essink-Bot ML, Krabbe PF, Bonsel GJ, et al. An empirical comparison of four generic health status measures. The Nottingham Health Profile, the Medical Outcomes Study 36-item Short-Form Health Survey, the COOP/WONCA charts, and the EuroQol instrument. Med Care 1997;35(5):522-37. [↑](#endnote-ref-4)
